# Fecal Colonization with Extended-Spectrum Beta-Lactamase and AmpC-Producing* Escherichia coli*

**DOI:** 10.1155/2016/3704150

**Published:** 2016-05-31

**Authors:** Mohamed H. Al-Agamy, Taghrid S. El Mahdy, Atef M. Shibl

**Affiliations:** ^1^Department of Pharmaceutics, Microbiology Division, College of Pharmacy, King Saud University, P.O. Box 2457, Riyadh 11451, Saudi Arabia; ^2^Department of Microbiology and Immunology, Faculty of Pharmacy, Al-Azhar University, Cairo, Egypt; ^3^Microbiology and Immunology Department, Faculty of Pharmacy, Helwan University, Cairo, Egypt; ^4^College of Medicine, Alfaisal University, Riyadh, Saudi Arabia

## Abstract

*Background*. Extended-spectrum *β*-lactamases (ES*β*Ls) and AmpC *β*-lactamases cause *β*-lactam resistance in* Escherichia coli*. Fecal colonization by ES*β*L- and/or AmpC-positive* E. coli* is a source of nosocomial infections.* Methods*. In order to investigate inpatient fecal colonization by ES*β*Ls and AmpC, antibiotic sensitivity tests were conducted and minimum inhibitory concentrations (MICs) were determined using the disk diffusion method and* E*-test, respectively. Characterization of ES*β*L and AmpC was performed using* E*-test strips, and a set of PCRs and DNA sequence analyses were used to characterize the ES*β*L and AmpC genes.* Results*. The whole collection of* E. coli* isolates (*n* = 50) was sensitive to imipenem, tigecycline, colistin, and fosfomycin, while 26% of the isolates showed reduced susceptibility to ceftazidime (MIC ≥ 4 *μ*g/mL). ES*β*L was phenotypically identified in 26% (13/50) of cases, while AmpC activity was detected in two ES*β*L-producing* E. coli* isolates. All ES*β*L-producing* E. coli* were positive for the CTX-M gene, eleven isolates carried *bla*_CTX-M-15_, and two isolates carried *bla*_CTX-M-14_ gene. Two CTX-M-positive* E. coli* isolates carried *bla*_CMY-2_.* Conclusions*. The alimentary tract is a significant reservoir for ES*β*L- and/or AmpC-producing* E. coli*, which may lead to nosocomial infection.

## 1. Introduction

A remarkable increase in fecal colonization rates with extended-spectrum beta-lactamase- (ES*β*L-), AmpC-plasmid mediated-, and/or carbapenemases-producing Enterobacteriaceae has been reported in many regions worldwide [1, 2]. Infections caused by Enterobacteriaceae, which are resistant to *β*-lactams, are coupled with the inappropriate use of antibiotics and/or a prolonged period of hospital admission. The rising use of carbapenems for empirical treatment of nosocomial infections has led to fast global dissemination of carbapenemase-positive* enterobacterial strains* [3]. ES*β*Ls arise through point mutations in TEM-1/TEM-2 and SHV-1. However, over the last three decades, non-TEM and non-SHV ES*β*Ls strains have been detected, primarily CTX-M. Enterobacteriaceae that produce CTX-M enzymes have shown rapid and concerning dissemination and have been documented as the most prevalent etiological infectious agents [4]. ES*β*L confers resistance to penicillins, cephalosporins, and monobactam (aztreonam), but they are susceptible to cephamycins (cefoxitin and cefotetan) and carbapenems (imipenem, meropenem, and doripenem) and are typically reserved by inhibitors of Ambler class A *β*-lactamase (clavulanic acid, tazobactam, or sulbactam). Most ES*β*Ls can hydrolyze fourth-generation cephalosporins. While AmpC *β*-lactamases confer resistance to penicillins, third-generation cephalosporins, monobactam, and cephamycins, they are sensitive to carbapenem and are not inhibited by *β*-lactamase inhibitors; however, they are inhibited by cloxacillin [5–7].

Numerous studies in Saudi Arabia have focused on the identification of ES*β*L-producing strains from clinical specimens [4], but there are few reports on the fecal colonization of ES*β*L-producing isolates in Saudi Arabia. Therefore, in the present study, we determine the incidence of ES*β*L- and/or AmpC cephalosporinase-producing* Escherichia coli* isolates in human fecal flora and investigated the genes encoding the corresponding enzymes.

## 2. Materials and Methods

### 2.1. Bacterial Identification

Fifty different* E. coli* isolates were isolated from 50 stool samples of different inpatients carriers, under nonoutbreak conditions, at a hospital in Riyadh, Saudi Arabia, from April 2014 to June 2014. Briefly, fresh stool specimens were aseptically collected and transported to the microbiology laboratory. Stool samples were suspended in sterile phosphate-buffered saline, pH 7. A 100 *μ*L volume was directly inoculated onto blood agar and Eosin Methylene Blue agar (Oxoid Microbiology Products, Hampshire, UK). After 48 h incubation at 37°C, the isolated organisms were identified by conventional procedures and automated identification systems with the API20E identification kit (bioMerieux, Marcy l'Etoile, France). These isolates were preserved in brain heart infusion broth containing 20% glycerol at −70°C.

### 2.2. Phenotypic Detection of ES*β*L

The isolates showing reduced susceptibility to ceftazidime (CAZ), cefotaxime (CTX), or aztreonam (ATM) (minimum inhibitory concentration (MIC) ≥ 1 *μ*g/mL or zone diameter ≤ 22 mm) were selected for screening of ES*β*L production (Clinical and Laboratory Standards Institute (CLSI), 2014).* E*-test ES*β*L strips were used in accordance with the manufacturer's instructions to evaluate ES*β*L production. The CAZ/ceftazidime + clavulanate- (CAZ/CAL-) ES*β*L* E*-test strip was used to detect ES*β*L production. The test is considered positive if the ratio of MIC of CAZ/CAL is ≥8. To inhibit AmpC *β*-lactamase, the CAZ/CAL-ES*β*L* E*-test was carried out on cloxacillin Mueller-Hinton agar, and the results were interpreted in a similar manner.

### 2.3. Phenotypic Detection of AmpC

The isolates showing reduced susceptibility to cefoxitin (FOX) or cefotetan (CTT) (zone diameter of 18 or 16 mm, resp.) were selected for screening of AmpC enzyme production [9]. The phenotypic detection test consists of a strip containing CTT on one end and CTT-cloxacillin (CTT/CXT) on the other end. Ratios of the MICs of CTT/CXT ≥ 8 are considered to indicate positive AmpC *β*-lactamase production.

### 2.4. Susceptibility Testing

MICs for the isolates showing a phenotype of producing ES*β*L and AmpC activities were determined by using* E*-test strips (bioMerieux, Marcy l'Etoile, France). Interpretation was based on the Clinical and Laboratory Standards Institute (CLSI) criteria [9].* Escherichia coli* ATCC 25922 strains were used as reference strains. The following antibiotics were tested: piperacillin (PIP), piperacillin/tazobactam (TZP), CAZ, CAZ/CAL, CTX, cefepime (FEP), ATM, FOX, CTT, CTT/CXT, imipenem (IMI), gentamicin (GM), amikacin (AK), ciprofloxacin (CI), colistin (COL), tigecycline (TGC), and fosfomycin (FOS).

### 2.5. Screening for the Presence of *β*-Lactamase Genes

The isolate was cultured in 2 mL of Tryptic Soy Broth (Difco, Franklin Lakes, NJ, USA). A 200 *μ*L volume of overnight culture was heated at 99°C in a heat block for 10 min. The obtained DNA was used in polymerase chain reaction (PCR) assays on a Techne Flexigene Thermal Cycler (Techne, Duxford, Cambridge, UK). Positive and negative controls were included in all PCR assays. All PCR products were analyzed on 0.8% agarose gels (incorporated with 0.5 mg/L ethidium bromide) and then visualized under UV light (Pharmacia LKB, Biotechnology AB, Gothenburg, Sweden) and photographed using a documentation system (CE, DP-CF-011.C, European Union).

The PCR primers used are listed in Table 1. The primers were used to search for class A *β*-lactamase genes (*bla*_TEM_, *bla*_SHV_, *bla*_OXA-1_, and *bla*_CTX-M_ families) and class C *β*-lactamase genes (*bla*_CMY_, *bla*_MOX_, *bla*_FOX_, *bla*_DHA_, *bla*_ACC_, *bla*_ACT_, *bla*_MIR_, *bla*_EBC_, *bla*_CIT_, and *bla*_BIL_). PCR assays were conducted as previously described [8].

### 2.6. Sequencing of *β*-Lactamase Genes

Purification of PCR amplicons was performed using a PCR purification kit (Qiagen, Hilden, Germany). PCR products of* bla* genes were sequenced on both strands using PCR primers to determine their molecular types. DNA sequences were analyzed using the ABI 3100 Genetic Analyzer (Applied Biosystems, Foster City, CA, USA) according to the manufacturer's recommendations.

## 3. Results

### 3.1. Bacterial Identification

Fifty fecal* E. coli* samples were isolated randomly from hospitalized patients in Riyadh, Saudi Arabia. The patients were treated for noninfectious diseases under nonoutbreak conditions.* Escherichia coli* isolates were identified manually and according to the API20E identification kit (bioMerieux, Marcy l'Etoile, France).

### 3.2. Characterization of *bla*_ES*β*L_ and *bla*_AmpC_

Thirteen of the 50* E. coli* isolates, which showed reduced susceptibility to CAZ, CTX, or ATM (MIC ≥ 1 *μ*g/mL or inhibition zone ≤ 22 mm), were selected for screening of ES*β*L and AmpC enzyme production using CAZ/CAL-ES*β*L and CTT/CXT-AmpC* E*-test strips. Thirteen isolates were positive for ES*β*L and two ES*β*L-positive isolates produced AmpC *β*-lactamase.

PCR was used to detect *bla*_ES*β*L_ genes and* AmpC* plasmid-mediated genes in ES*β*L- and AmpC-positive* E. coli* isolates (*n* = 13). The results of PCR and DNA sequencing of* bla* genes are shown in Table 2.

Eleven of 13 ES*β*L-producing* E. coli* isolates were found to contain CTX-M-15, while two isolates harbored *bla*_CTX-M-14_. CMY-2-positive isolates (*n* = 2) were concomitant with CTX-M-15. All ES*β*L-producing* E. coli* isolates (*n* = 13) were positive for *bla*_TEM-1_, while eight (61.5%) isolates carried *bla*_OXA-1_. In contrast, three (23%) isolates were found to contain *bla*_SHV-1_.

### 3.3. Antimicrobial Resistance Pattern of ES*β*L and AmpC Enzyme-Positive Isolates

The MICs of 17 antimicrobial agents were determined for* E. coli* fecal isolates. The results of the susceptibility pattern for ES*β*L and AmpC enzyme-producing* E. coli* isolates are illustrated in Table 2.

## 4. Discussion

The human and animal alimentary tracts are vital reservoirs for ES*β*L-, carbapenemases-, and AmpC enzyme-producing Enterobacteriaceae. Patient-to-patient transmission of resistant microorganisms may occur in hospitals [10, 11]. The overuse of antibiotics has recently been associated with the emergence of resistant intestinal bacteria, particularly ES*β*L-, carbapenemases-, and AmpC enzyme-producing Enterobacteriaceae. Numerous studies have demonstrated that exposure to *β*-lactam antibiotics is a risk factor for the selection of multidrug-resistant* E. coli* [12]. Therefore, the current study examined the antimicrobial resistance patterns of fecal* E. coli* isolates, as well as the molecular basis for their *β*-lactam resistance mechanisms, using phenotypic and genotypic methods. The present study included 50 patients admitted to a hospital in Riyadh, Saudi Arabia, from April 2014 to June 2014. These patients were treated for noninfectious diseases under nonoutbreak conditions. Fifty fecal stool specimens collected from the 50 patients were cultured on blood agar and EMB agar as described in Materials and Methods. Fifty suspected* E. coli* isolates were selected and the isolates were identified by conventional procedures and using the API20E identification kit. All isolates were identified as* E. coli*.

Production of *β*-lactamases is the main mechanism of *β*-lactam resistance in Gram-negative bacteria, including* E. coli* [6, 7]. Several *β*-lactamases (ES*β*Ls, AmpC enzymes, and carbapenemases) have been previously reported in fecal* E. coli* isolates [13–15].

In the present study, 100% of 50* E. coli* isolates were found to be sensitive to imipenem and 13 (26%) of 50 isolates were resistant or showed reduced susceptibility to CAZ, CTX-M, or ATM. The carbapenem susceptibility results indicated that our isolates did not harbor carbapenemase, while 26% (13/50) of the* E. coli* isolates harbored ES*β*L and/or AmpC *β*-lactamase. Two of 13 isolates exhibited reduced susceptibility to cephamycins (FOX and CTT).

Phenotypic screening for the presence of different types of *β*-lactamases was conducted using* E. coli* isolates. Thirteen* E. coli* isolates were selected for screening of ES*β*L and AmpC *β*-lactamase production using CAZ/CAL-ES*β*L, and CTT/CXT-AmpC* E*-test strips were used to detect ES*β*L production. Using phenotypic detection methods, all isolates were found to produce ES*β*L, while two isolates phenotypically produced AmpC enzyme. A battery of PCR assays was conducted to detect *bla*_ES*β*L_ genes and AmpC plasmid-mediated genes in the 13* E. coli* isolates. Therefore, class A and class C *β*-lactamase genes were tested. The PCR-purified product was subjected to DNA sequencing to identify the gene variants. The results of molecular characterization of* bla* genes are shown in Table 2. PCR amplification and DNA sequencing analyses of the PCR products showed that all isolates possessed a CTX-M-type ES*β*L and that *bla*_CTX-M-15_ was present in 1 isolate, while two isolates contained *bla*_CTX-M-14_. Other CTX-M families were not detected. The gene encoding CMY-2 enzyme was detected in two* E. coli* isolates. CMY-2-positive isolates are concomitant with CTX-M-15. All* E. coli* isolates (*n* = 13) were positive for *bla*_TEM-1_, while 61.5% and 23% of the isolates contained *bla*_OXA-1_ and *bla*_SHV-1_, respectively. The increase in expression of the AmpC *β*-lactamases may mask the recognition of ES*β*Ls [16]. Therefore, in the present study, the genotypic methods revealed that all 13 strains were ES*β*L CTX-M-positive, while phenotypic methods showed that 11 strains were ES*β*L-positive and two strains were AmpC enzyme-positive. AmpC-producing strains producing CTX-M-15 may act as a dormant reservoir for ES*β*Ls.

Numerous studies have documented a remarkable increase in intestinal colonization rates with ES*β*L- and AmpC enzyme-producing Enterobacteriaceae in many countries [2, 10, 11, 14–17]. The prevalence of ES*β*L-producing* E. coli* fecal isolates varies widely from country to country, from region to region, and at different time periods. A high incidence of fecal carriage rate of ES*β*L-producing* E. coli* has been observed in Asia, Africa, and South America [13, 14, 18–21], while a significantly lower prevalence of ES*β*L-producing* E. coli* fecal isolates was reported in most European countries [22, 23]. In Argentina, the rate of fecal carriage of Enterobacteriaceae-resistant strains to third-generation cephalosporins was 26.8% [20]. Villar et al. [20] reported that 20.22% and 6.7% of fecal strains were colonized by ES*β*L- and AmpC enzyme-producing Enterobacteriaceae [20]. In Egypt, Al-Agamy et al. reported that 22.6% and 3.22% of hospitalized patients were colonized by ES*β*L- and AmpC-positive* E. coli*, respectively. The *bla*_CTX-M_-like gene was the predominant ES*β*L gene, detected in 71.4% of ES*β*L-producing* E. coli* isolates [21]. In a recent study in Egypt, Bassyouni et al. reported that 21% and 3% of patients were colonized by ES*β*L- and AmpC-producing* E. coli*, respectively. They also found that *bla*_SHV_ gene was the predominant ES*β*L gene, detected in 81.8% of the resistant* E. coli* isolates [13]. In Korea, 20.3% of fecal Enterobacteriaceae members were ES*β*Ls [19]. In India, the prevalence of ES*β*L-positive* E. coli* isolates was 19% in healthy volunteers from the community [14]. In Libya, 13.4% and 6.7% of* E. coli* isolates were ES*β*Ls- and AmpC-positive, respectively [18]. In a previous study in Saudi Arabia, 17.7% of strains were found to be ES*β*L-positive [24]. A high (26.1%) prevalence was detected in inpatients, followed by outpatients (15.4%), and the lowest prevalence rate (13.1%) was detected in healthy individuals [24]. In the present study, the prevalence of ES*β*L-producing* E. coli* was 26%. Despite differences in the date and region of isolation, the prevalence of fecal carriage rate of ES*β*L in the present study (26%) was in agreement with the prevalence (26.1%) reported by Kader et al. In contrast, the prevalence rate of ES*β*L-producing Enterobacteriaceae was 2.9% among healthy Swedish children.* Escherichia coli* containing CTX-M *β*-lactamase predominated, and only one* E. coli* isolate harbored genes encoding for CMY [22]. The carriage rate of ES*β*L- and AmpC enzyme-producing* E. coli* was 3.57% and 2.38% among 84 Danish army recruits, respectively. *bla*_CTX-M-14_ gene was the predominant ES*β*L gene detected in three (100%) ES*β*L-producing* E. coli* isolates, while *bla*_CMY-2_ was detected in two AmpC enzyme-producing* E. coli* isolates [23]. In Spain, the prevalence of ES*β*L and AmpC enzyme carriers was 5.06% and 0.59%, respectively [25]. *bla*_CTX-M_ genes were the ES*β*L dominating genes (96.15%) and CTX-M-14 was the most prevalent gene (50%), followed by CTX-M-15 (40%). CMY-2 was the most prevalent gene (81.25%), followed by DHA-1 (18.75%) [25].

MICs were determined for ES*β*L- and AmpC enzyme-producing* E. coli* (*n* = 13) isolates. The results of MIC are shown in Table 2.

In conclusion, a high incidence of carriage of ES*β*L-positive* E. coli* fecal isolates among hospitalized patients in Riyadh was detected, reaching 26%, with *bla*_CTX-M-15_ (84.6%) being the most predominant gene. The emergence of fecal carriage of CMY-2-producing* E. coli* among hospitalized patients has been reported to be 4%. These outcomes emphasize the importance of the intestinal tract as a reservoir for ES*β*L- and AmpC enzyme-producing* E. coli*, which may lead to nosocomial infection. The admission of colonized fecal carriers of ES*β*L- and AmpC-positive* E. coli* to the medical setting increases the possibility of other patients acquiring infection in the same hospital. Our results emphasize the necessity for continuous surveillance in hospitals to detect the ES*β*L-, AmpC enzyme-, and carbapenemase-producing strains and multidrug strains as well applying effective strategies for antimicrobial therapy and infection control measures to decrease the abuse and misuse of antimicrobial agents against resistant strains and to prevent their spread.

## Figures and Tables

**Table 1:** Primers used for amplification of the tested *β*-lactamase genes (Dallenne et al., 2010 [8]).

PCR type	Target	Primer	Sequence of primers (5′-3′)	Amplified products (bp)
Multiplex I TEM, SHV, and OXA-1-like	TEM	MultiTSO-T_for	CATTTCCGTGTCGCCCTTATTC	800
		MultiTSO-T_rev	CGTTCATCCATAGTTGCCTGAC	
	SHV	MultiTSO-S_for	AGCCGCTTGAGCAAATTAAAC	713
		MultiTSO-S_rev	ATCCCGCAGATAAATCACCAC	
	OXA-1	MultiTSO-O_for	GGCACCAGATTCAACTTTCAAG	564
		MultiTSO-O_rev	GACCCCAAGTTTCCTGTAAGTG	

Multiplex II CTX-M group 1, group 2, and group 9	CTX-M group 1	MultiCTXMGp1_for	TTAGGAARTGTGCCGCTGYA	688
		MultiCTXMGp1-2_rev	CGATATCGTTGGTGGTRCCAT	
	CTX-M group 2	MultiCTXMGp2_for	CGTTAACGGCACGATGAC	404
		MultiCTXMGp1-2_rev	CGATATCGTTGGTGGTRCCAT	
	CTX-M group 9	MultiCTXMGp9_for	TCAAGCCTGCCGATCTGGT	561
		MultiCTXMGp9_rev	TGATTCTCGCCGCTGAAG	

CTX-M group 8/25	CTX-M group 8/25	CTX-Mg8/25_for	AACRCRCAGACGCTCTAC	326
		CTX-Mg8/25_rev	TCGAGCCGGAASGTGTYAT	

Multiplex III ACC, FOX, MOX, DHA, CIT, and EBC	ACC-1 and ACC-2	MultiCaseACC_for	CACCTCCAGCGACTTGTTAC	346
		MultiCaseACC_rev	GTTAGCCAGCATCACGATCC	
	FOX-1 to FOX-5	MultiCaseFOX_for	CTACAGTGCGGGTGGTTT	162
		MultiCaseFOX_rev	CTATTTGCGGCCAGGTGA	
	MOX-1, MOX-2, CMY-1, CMY-8 to CMY-11, and CMY-19	MultiCaseMOX_for	GCAACAACGACAATCCATCCT	895
		MultiCaseMOX_rev	GGGATAGGCGTAACTCTCCCAA	
	DHA-1 and DHA-2	MultiCaseDHA_for	TGATGGCACAGCAGGATATTC	997
		MultiCaseDHA_rev	GCTTTGACTCTTTCGGTATTCG	
	LAT-1 to LAT-3, BIL-1, CMY-2 to CMY-7, CMY-12 to CMY-18, and CMY-21 to CMY-23	MultiCaseCIT_for	CGAAGAGGCAATGACCAGAC	538
		MultiCaseCIT_rev	ACGGACAGGGTTAGGATAGY	
	ACT-1 and MIR-1	MultiCaseEBC_for	CGGTAAAGCCGATGTTGCG	683

**Table 2:** Antimicrobial susceptibility profiles of ESβL- and AmpC-producing *E. coli* isolates from fecal samples and associated resistance patterns.

Isolates number	*E*-test MIC (mg/L)																	Resistance genes
	PIP	TZP	CTX	CAZ	CAZ/CAL	FEP	ATM	FOX	CTT	CTT/CXT	IMI	GM	AK	CI	COL	TGC	FOS	
EC1	>256	<1	>256	32	32/2	4	8	0.032	0.25	<0.5/<0.5	0.06	0.5	2	1	<0.016	0.25	0.016	TEM-1+CTX-M-15+OXA-1
EC2	>256	<1	>256	16	16/0.5	4	16	0.065	0.25	<0.5/<0.5	0.12	1.5	3	0.25	<0.016	0.25	<0.016	TEM-1+CTX-M-15++SHV-1
EC3	>256	64	>256	>256	>32/4	12	>256	0.125	0.25	<0.5/<0.5	0.25	64	3	4	<0.016	0.125	<0.016	TEM-1+CTX-M-15+OXA-1
EC4	>256	8	>256	32	32/0.064	8	16	0.032	0.06	<0.5/<0.5	0.06	12	4	4	<0.016	0.125	<0.016	TEM-1+CTX-M-15+OXA-1
EC5	>256	32	>256	16	16/0.5	4	12	0.125	0.125	<0.5/<0.5	0.03	8	3	6	<0.016	0.25	<0.016	TEM-1+CTX-M-15+SHV-1
EC6	>256	8	>256	>256	>32/4	192	>256	0.25	0.25	<0.5/<0.5	0.06	192	48	>32	<0.016	0.25	<0.016	TEM-1+CTX-M-15+OXA-1
EC7	>256	8	>256	>256	>32/4	128	192	0.25	0.25	<0.5/<0.5	0.12	128	16	>32	<0.016	0.25	<0.016	TEM-1+CTX-M-15+OXA-1
EC8	>256	<1	>256	>256	>32/4	96	128	0.25	0.25	<0.5/<0.5	0.06	96	0.5	0.25	<0.016	0.25	0.125	TEM-1+CTX-M-14+OXA-1
EC9	>256	16	>256	4	4/0.064	3	3	0.032	0.032	<0.5/<0.5	0.06	8	2	0.25	<0.016	0.38	<0.016	TEM-1+CTX-M-15
EC10	>256	4	>256	6	6/0.125	4	6	0.032	0.032	<0.5/<0.5	0.125	16	1	0.5	<0.016	0.25	<0.016	TEM-1+CTX-M-15+SHV-1
EC11	>256	<1	>256	48	>32/1	8	24	0.032	0.032	<0.5/<0.5	0.25	2	32	1	<0.016	0.25	<0.016	TEM-1+CTX-M-14+OXA-1
EC12	>256	>256	>256	>256	>32/>4	48	>256	64	32	32/32	0.25	>256	192	0.75	<0.016	0.25	0.016	TEM-1+CTX-M-15+CMY-2+OXA-1
EC13	>256	128	>256	>256	>32/>4	64	>256	64	48	>32/>32	0.25	128	4	>32	<0.016	0.38	0.016	TEM-1+CTX-M-15+CMY-2

PIP: piperacillin; TZP: piperacillin/tazobactam; CAZ: ceftazidime; CAZ/CAL: ceftazidime/ceftazidime + clavulanic acid; CTX: cefotaxime; FEP: cefepime; ATM: aztreonam; FOX: cefoxitin; CTT: cefotetan; CTT/CXT: cefotetan/cefotetan + cloxacillin; IMI: imipenem; GM: gentamicin; AK: amikacin; CI: ciprofloxacin; COL: colistin; TGC: tigecycline; FOS: fosfomycin.
